# High-Temperature Conditions Promote Soybean Flowering through the Transcriptional Reprograming of Flowering Genes in the Photoperiod Pathway

**DOI:** 10.3390/ijms22031314

**Published:** 2021-01-28

**Authors:** Dong Hyeon No, Dongwon Baek, Su Hyeon Lee, Mi Sun Cheong, Hyun Jin Chun, Mi Suk Park, Hyun Min Cho, Byung Jun Jin, Lack Hyeon Lim, Yong Bok Lee, Sang In Shim, Jong-Il Chung, Min Chul Kim

**Affiliations:** 1 Division of Applied Life Science (BK21 Four), Gyeongsang National University, Jinju Daero 501, Jinju 52828, Korea; no0513w@naver.com (D.H.N.); leesuhyeon86@gmail.com (S.H.L.); hmcho86@gmail.com (H.M.C.); scv5789@naver.com (B.J.J.); dlafkrgus@gnu.ac.kr (L.H.L.); yblee@gnu.ac.kr (Y.B.L.); 2 Plant Molecular Biology and Biotechnology Research Center, Gyeongsang National University, Jinju Daero 501, Jinju 52828, Korea; dw100@hanmail.net (D.B.); misugip@hanmail.net (M.S.P.); 3 Institute of Agriculture & Life Science, Gyeongsang National University, Jinju Daero 501, Jinju 52828, Korea; mscheong@gnu.ac.kr (M.S.C.); hj_chun@hanmail.net (H.J.C.); sishim@gnu.ac.kr (S.I.S.); jongil@gnu.ac.kr (J.-I.C.)

**Keywords:** soybean, high temperature, carbon dioxide, flowering time, gene expression

## Abstract

Global warming has an impact on crop growth and development. Flowering time is particularly sensitive to environmental factors such as day length and temperature. In this study, we investigated the effects of global warming on flowering using an open-top Climatron chamber, which has a higher temperature and CO_2_ concentration than in the field. Two different soybean cultivars, Williams 82 and IT153414, which exhibited different flowering times, were promoted flowering in the open-top Climatron chamber than in the field. We more specifically examined the expression patterns of soybean flowering genes on the molecular level under high-temperature conditions. The elevated temperature induced the expression of soybean floral activators, *GmFT2a* and *GmFT5a* as well as a set of *GmCOL* genes. In contrast, it suppressed floral repressors, *E1* and *E2* homologs. Moreover, high-temperature conditions affected the expression of these flowering genes in a day length-independent manner. Taken together, our data suggest that soybean plants properly respond and adapt to changing environments by modulating the expression of a set of flowering genes in the photoperiod pathway for the successful production of seeds and offspring.

## 1. Introduction

Since 1850, which can also be referred to as the industrial era, the effects of global warming have been accelerating, and the Earth’s surface temperature has been heating up [1]. Global warming causes extreme weather, which threatens crop growth and productivity. This includes more intense precipitation with less rainfall, a higher incidence of drought, and increased extreme temperature fluctuations, all of which may negatively affects agricultural production [2,3]. According to the intergovernmental panel on global warming (IPCC; http://www.ipcc.ch/repor/sr15), the average surface temperature has increased by 1.04 °C over the last five years (2014–2018), and the largest cause of changes in Earth’s surface temperature is elevated atmospheric CO_2_ [4,5]. These environmental factors cause global warming and are known to be major constraints to crop adaptation and productivity, as they affect plant growth and development [6,7]. High-temperature conditions induces pre-maturation such as early flowering with less photosynthetic activity [7,8], and elevated CO_2_ concentration is highly related with the CO_2_ usage of plants when regulating between photosynthesis and photorespiration [9].

Soybean (*Glycine max*), a facultative short-day (SD) plant, is one of the most extensively cultivated and consumed crops in the world [10], since soybean serves not only as a good source of protein and oil for the human diet and livestock feeding, but also as a biofuel [11,12]. Soybean accessions from different geographical areas exhibit extensive genetic diversity and these genetic variations are highly associated with agronomic traits [13]. For example, soybean flowers, in response to the photoperiod, change from long-day (LD) to floral-inductive short-day (SD) conditions. However, soybean demonstrates a wide range of latitude adaptability that has evolved or developed into different variations to control the timing of flowering under various day length conditions [14].

Photoperiod is the term for the daily cycle of day and night and plays a role as an environmental signal that affects flowering. According to flowering response, plants are classified into three classes, LD, SD, and day-neutral plants, which flower depending on the photoperiod in every 24-h duration [15]. From studies using *Arabidopsis* as a model plant, a consensus mechanism underlying flowering time control has been identified, with highly conserved molecular components among flowering plant species [15]. GIGANTEA (GI) acts as a master regulator that transmits the circadian signal to the flowering regulator CONSTANS (CO) and FLOWERING LOCUS T (FT) [16]. More specifically, *FT* genes encode a systemic signaling molecule, florigen, which is synthesized in the leaves and moves to the apex to induce flowering [17]. CO is a transcriptional activator and is regulated on the transcription and post-translational level by daylight and plays an important role for *FT* transcription [18]. GI is a plant-specific nuclear protein that does not have any known functional domains [16]. The GI-CO-FT module is the main photoperiod pathway in *Arabidopsis* [16,19].

In spite of the emerging variability in flowering times depending on the cultivar, most soybeans promote flowering in response to SD and suppress flowering under LD conditions by modulating various flowering gene activities, such as *E* loci, as well as soybean *FT* and *CO-Like* (*COL*), soybean orthologs of the major *Arabidopsis* flowering genes [14,20,21]. *E* loci function in flowering and maturity has been reported in soybean plants [20,22]. For example, *E1* and *E2* were recently cloned and identified as floral repressors, which contribute to a late flowering phenotype under LD [23]. *E1* has the largest effect on soybean flowering during photoperiodic regulation by repressing flowering under LD conditions [20]. *E1* is a legume-specific gene, with no homologue in *Arabidopsis* or rice, which is predicted as a transcription repressor of the B3 superfamily [24]. *E2* encodes GmGI2 [14], a homolog of the *Arabidopsis GI* gene that functions as a key regulator of photoperiodic flowering [25].

*FT* is conserved among plant species, and 10 soybean *FT* homologs, *GmFTs*, have been reported [20]. *GmFT2a*, identified as the *E9* locus gene [26], and *GmFT5a* promote flowering by photoperiod response [27]. Photoperiodic regulation of *GmFTs* expression is controlled at the downstream of *E1* and *E2* [23]. Different from *Arabidopsis* CO, which induces *FT* expression depending on regulatory modules of light signaling and the circadian clock [28], soybean *COL* genes, *GmCOLs*, exhibit differential profiles and may regulate flowering differentially despite of high sequence homology to *Arabidopsis* CO [29]. Soybean mutant plants, which have a mutation in the *GmCOL1a* gene, show early flowering under LD conditions, and the overexpression of *GmCOL1a* exhibits late flowering [30]. E1 and E2 influence the expression of *GmCOL1a* and vice versa [30]. However, this feedback loop has not been fully elucidated, thus far.

In this study, we investigated the effects of global warming using an open-top Climatron chamber, which mimics an environment with an elevated CO_2_ concentration and higher temperature, on soybean growth and flowering. Although the increased CO_2_ concentration contributed to an increase in photosynthesis efficiency and enhanced yields, the elevated temperature promoted flowering in both early and late flowering soybean cultivars. In addition, we firstly confirmed that the high temperature promoted flowering by modulating the expression of signaling components in a photoperiod-dependent flowering pathway in soybean plants. As predicted from the early flowering phenotype under Climatron conditions, floral activator genes, including *GmFT2a* and *GmFT5,* were induced in response to high temperature, whereas the expression of their upstream negative regulators, *E1* and *E2,* was suppressed under both SD and LD conditions. Moreover, we also provided the first evidence supporting the involvement of some members of the *GmCOLs* gene family in high temperature-induced early flowering in soybean plants.

## 2. Results

### 2.1. Climatron Condition Affects Soybean Growth and Development Such as Flowering

To investigate the effects of global warming, including elevated temperature and CO_2_ concentration, on soybean growth and development, we grew two soybean cultivars, Williams 82 and IT153414, in an open-top Climatron chamber (Figure S1). The physiological traits, such as vegetative growth and flowering time, of the two cultivars were compared with samples grown in the field (Figures S2 and S3). The Williams 82 cultivar, which was used as a reference genome sequence [31], demonstrates early flowering, and IT153414 is a late flowering cultivar. The temperature in the Climatron was programmed to be 3 °C to 4 °C higher than the local field daytime temperature (Figure S2a). The level of CO_2_ was also programmed to be 200 ppm higher than local-field conditions (Figure S2b). These values were based on a record of the temperature and CO_2_ concentration taken at 11 a.m. each day during cultivation between 2019 and 2020. Both cultivars showed improved vegetative growth when grown in the Climatron (Figure S2c,d).

To analyze soybean growth and development under Climatron conditions in more detail, we measured the height when growth stopped and leaf senescence such as yellowing showed. As shown Figure S2, both cultivars grown in the Climatron were taller than those in the field (Figure S3a). As photosynthesis is a biochemical pathway that converts CO_2_ in the air to sugars, it contributes to physiological plant growth and development such as vegetative tissue expansion by capturing light energy [32]. We examined the change in photosynthesis efficiency (F_v_/F_m_) on the middle leaflet of the youngest trifoliate leaves from each plant (*n* = 10) at the V7 stage, in which vegetative growth is beginning on the seventh trifoliate leaves (https://webapp.agron.ksu.edu/agr_social/m_eu_article.throck?article_id=1286). Both the Williams 82 and IT153414 cultivars in the Climatron showed higher F_v_/F_m_ than in the open field (Figure S3b).

We further examined agronomic traits related to crop yield and quality, such as pod number, seed number, and 100-seed weight, from the Climatron and field environments. Since soybean seeds are produced and matured in pods, pod number is an important agronomic characteristic. The pod number is determined before floral initiation due to the potential sites of flowering branches related with the leaf numbers and leaf axils. Furthermore, pod density and/or seed density are fully determined just after flowering [33]. To ascertain these agronomic results, we counted the number of pods and seeds from each plant and measured the 100-seed weight per plant grown in the Climatron and field when harvesting matured seeds of the Williams 82 and IT153414 cultivars, which have different harvest seasons due to different flowering times and maturation seed periods. Both soybean cultivars grown in the Climatron revealed higher pod numbers and more seeds the field-grown plants (Figure S3c,d). Moreover, the 100-seed weight of the Williams 82 cultivar showed similar values for both growth conditions, while the IT153414 plants from the Climatron showed lower values than the field-grown equivalent (Figure S3e). This observation indicated that the seed yield of soybean plants has a positive relationship with pod number and seed number under high efficiency photosynthesis conditions. In addition, the 100-seed weight indicated that seed size is similar or smaller when growing in the Climatron as compared to normal field conditions. These results demonstrated that the Climatron environment contributes to a better yield in soybean plants.

As shown in vegetative growth (Figures S2 and S3), Climatron conditions also affected the flowering developmental process (Figure S3f). The Williams 82 cultivar developed its first flower at 42 days after sowing (DAS) in the field and 39 DAS in the Climatron. Similarly, IT153414 produced flowers at 63.5 DAS in the field and 59 DAS in the Climatron (Figure S3f). This result indicates that a Climatron environment promotes flowering. Furthermore, a Climatron-mediated early-flowering phenotype was observed in both the Williams 82 and IT153414 cultivars.

### 2.2. High-Temperature Conditions Regulated Expression of Soybean Flowering Genes in Photoperiod Pathway

We found that the environment in the open-top Climatron chamber, in which two different factors were controlled (elevated CO_2_ concentration and temperature), as compared to field conditions, resulted in an early-flowering phenotype in genetically different soybean cultivars with respect to early or late flowering. On the basis of the previous reports showing that elevated CO_2_ concentration delayed the occurrence of the first open flower in soybean [34], pigeon pea [35], rice [36], and annual grasses [37] by one to seven days depending on species, we hypothesized that higher ambient temperature in the Climatron induces early-flowering phenotype. In addition, we examined the expression patterns of soybean flowering genes under high-temperature conditions.

To test the effects of high temperature on the expression of soybean flowering genes, we grew Williams 82 and IT153414 cultivars in growth chamber at 20 °C (control) and 30 °C (high temperature) under both SD and LD until the V1 stage, in which vegetative growth begins on the first trifoliate leaves. We harvested the first trifoliate leaves from three independent seedlings in each growth condition and extracted total RNAs. Primarily, we analyzed the expressions of the *GmFT2a* and *GmFT5a* genes, which play a crucial role in promoting soybean flowering [17,25,35].

**Figure 1:**
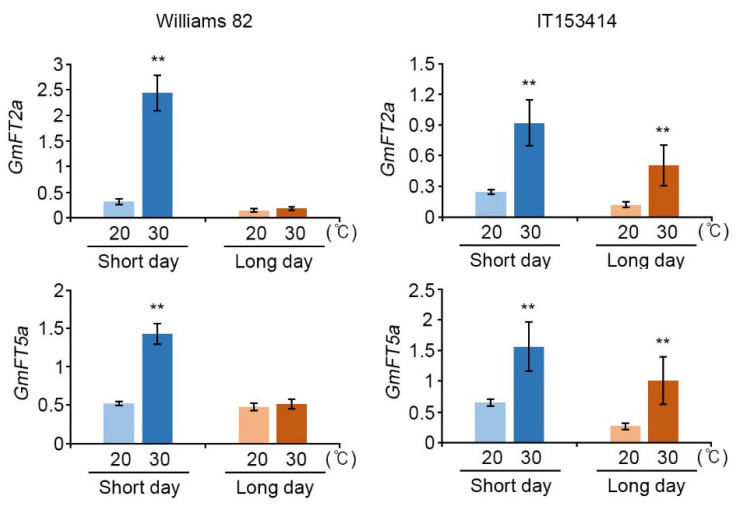
The expression of *GmFT2a* and *GmFT5a* in Williams 82 and IT153414 cultivars under high-temperature conditions. Total RNAs were extracted from the first trifoliate leaves at the V1 stage of soybeans grown in a growth chamber at 20 °C (bright color) and 30 °C (dark color) in short-day (SD, 12 h Light/12 h Dark, Blue) and long-day (LD, 16 h Light/8 h Dark, Orange) conditions. The relative transcript level of *GmFT2a* and *GmFT5a* was analyzed by quantitative real-time PCR (qRT-PCR). The expression of genes was normalized to that of *GmPPB2*. The values are means ± SD of three biological replicates (*n* = 3 for each replicate) with three technical replicates each. Asterisks represent significant differences in high temperature (30 °C, dark color) from the relative value of control temperature (20 °C, bright color) (*, *p* < 0.05; **, *p* < 0.01; Student’s *t*-test).

We confirmed that high temperature significantly induced the expression of these floral activators, *GmFT2a* and *GmFT5a,* under both SD and LD conditions, except for LD-grown Williams 82 plants (Figure 1).

**Figure 2:**
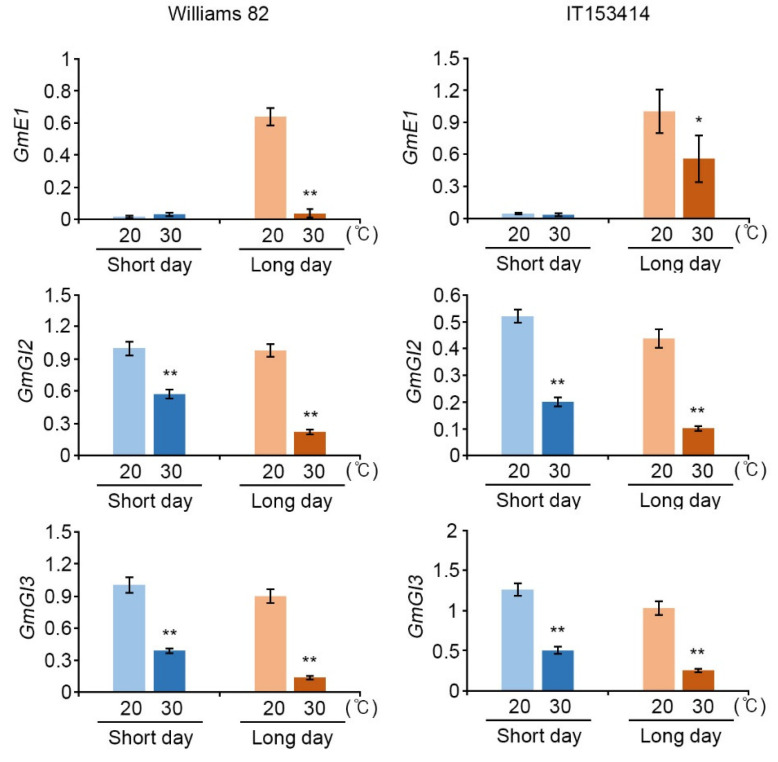
The gene expression of soybean *E1* and *E2* homologs in Williams 82 and IT153414 cultivars under high-temperature conditions. Two soybean cultivars, Williams 82 and IT153414, were grown at control (20 °C, bright color) and high temperature (30 °C, dark color) under SD (12 h Light/12 h Dark, Blue) and LD (16 h Light/8 h Dark, Orange) conditions. The relative transcript level of *GmE1*, *GmGI2*, and *GmGI3* was analyzed by qRT-PCR. The expression of *GmPPB2* was used for normalization. The quantitative values represent means ± SD (*n* = 3) and qRT-PCR replicated three times with similar results. Asterisks represent significant difference between high temperature (30 °C, dark color) and control temperature (20 °C, bright color) (*, *p* < 0.05; **, *p* < 0.01; Student’s *t*-test).

Next, we further tested the expression of floral repressor genes including *E1* and *E2* homologs (*GmGI1*, *GmGI2*, and *GmGI3*), which play roles in suppressing soybean flowering in LD by repressing the expression of the *GmFT2a* and *GmFT5a* genes under floral non-inductive LD conditions [23]. As expected, all tested floral repressor genes, including *E1*, *E2* (*GmGI2),* and its homolog, *GmGI3*, were down-regulated under the high-temperature condition in both cultivars, Williams 82 and IT153414 (Figure S4). To confirm this result, we further performed qRT-PCR and showed that the expression of all floral repressor genes, which have different molecular functions, were suppressed by high-temperature conditions in both the Williams 82 and IT153414 cultivars (Figure 2). The transcript level of *E1*, a critical photoperiodic responsive gene, was rarely detected under the SD condition but was much more prevalent under the LD condition, evidencing its specific role in LD (Figure 2) [20]. Although high-temperature conditions did not affect *E1* expression under SD, *E1* expression was reduced under LD and its suppression by high temperature was more drastic in the early-flowering cultivar, i.e., Williams 82, than the IT153414 cultivar (Figure 2). In addition, transcript levels of other repressors, *E2 (GmGI2*) and its homolog, *GmGI3,* were also suppressed under both SD and LD by high temperature (Figure 2). These results indicate that high temperature suppresses the expression of floral repressor genes, *E1* and *E2* homologs, *GmGI2* and *GmGI3*, in a day length-independent manner.

**Figure 3:**
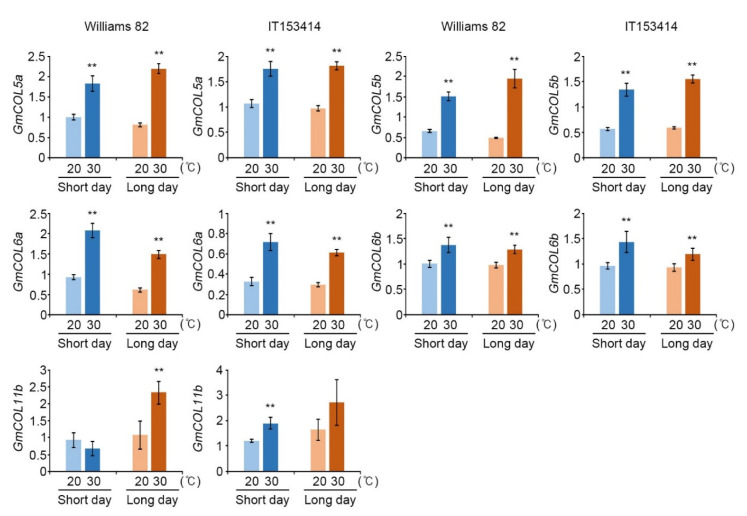
The expression of soybean *CO-Like* (*GmCOL*) genes under high-temperature conditions. Total RNAs were extracted from the first trifoliate leaves at the V1 stage of soybean cultivars grown at control (20 °C, bright color) and high temperature (30 °C, dark color) in short-day (SD, 12 h Light/12 h Dark, Blue) and long-day (LD, 16 h Light/8 h Dark, Orange) chambers. The relative transcript levels of *GmCOL5a*, *GmCOL5b*, *GmCOL6a*, *GmCOL6b*, and *GmCOL11b* were analyzed by qRT-PCR. Expression of genes was normalized to that of *GmPPB2*. Values are means ± SD from three biological replicates with three technical replicates each. The qRT-PCR was replicated biologically three times with similar results. Asterisks represent significant differences between high temperature (30 °C, dark color) and control temperature (20 °C, bright color) (*, *p* < 0.05; **, *p* < 0.01; Student’s *t*-test).

In *Arabidopsis*, *CO* is a central regulator for *FT* expression in a photoperiodic pathway [38]; although there are 26 annotated *GmCOL* genes in a soybean genome [29,39], their regulatory role in *FT* expression has been only reported by case studies of *GmCOL1a*/1*b* and *GmCOL2a*/2*b,* suggesting that legume *COL* genes have different regulatory roles from *Arabidopsis* CO [29,30]. For example, overexpression of *GmCOL1a* in soybean down-regulates *GmFT2a* and *GmFT5a* under LD conditions, but up-regulates under SD conditions [30], and the expression of *GmCOL1a*/1*b* and *GmCOL2a*/2*b* fully complemented the *co-1 Arabidopsis* mutant phenotype to activate *FT* expression [29]. The roles of most of *GmCOL* genes in flowering determination or controlling target gene expression, including *GmFT2a* and *GmFT5a,* are not understood clearly yet. Thus, we investigated the expression of all known 26 *GmCOL* genes to understand their responses to high temperature, and we observed the expression of *GmCOL5a/5b* and *GmCOL6a/6b* genes was up-regulated in both cultivars as well as in both day length conditions in response to the high temperature (Figure S5). Furthermore, *GmCOL11b* was up-regulated by high temperature despite its expression level being lower than *GmCOL5a/5b* and *GmCOL6a/6b* (Figure S5). In addition, we confirmed this result by qRT-PCR (Figure 3).

Taken together, our results suggest that high temperature promotes soybean flowering by differentially regulating the expression of major flowering genes in a photoperiodic pathway. In detail, high-temperature conditions suppressed floral repressors, i.e., the *E1* and *E2* family genes, and in contrast, it activated floral activators, *GmFT2a* and *GmFT5a* as well as the *GmCOL5a/5b* and *GmCOL6a/6b* genes. Moreover, it is also suggested that high temperature promotes flowering in a day length-independent manner, indicating that the effect of high temperature on soybean flowering overrides the photoperiod-dependent flowering time control.

## 3. Discussion

Global warming directly and indirectly affects crop production. Global warming is a long-term challenge that requires urgent action to fulfill the gaps in knowledge in order to develop strategies for preventing intensive change. There are three key global change factors associated with global warming: Rising levels of greenhouse gases (CO_2_, O_3_, and CH_4_), rising temperatures, and reduced water availability [40]. In this study, we primarily observed soybean growth according to two common factors: An elevated concentration of atmospheric CO_2_, which is the major greenhouse gas and is useful to plants, and high temperature.

Elevated temperatures affect the developmental and physiological plant processes that result in crop yield and quality [7,41]. Heat stress during legume reproduction causes significant loss of seed yield resulting from a decreased number of seeds, owing to the reduction in pod numbers and seeds per a pod [42,43,44]. Interestingly, we observed better crop yields from soybean under high-temperature conditions (Figure S3), which suggests that maintenance of photosynthesis (Figure S3b) due to a higher concentration of CO_2_ (Figure S2) may reduce the negative impacts on growth and seed maturation caused by high-temperature conditions, as well as enhancing adaptability to the high temperatures. It is evident that high temperatures inhibit photosynthetic carbon gain in crop plants [7,45]. For example, in field-grown soybean, a 3.5 °C increase above ambient seasonal growth temperatures results in decreased photosynthesis and carbon assimilation [45,46]. Although studies on the effects of high temperature on sugar content in legume reproductive organs is scarce, there is supporting evidence that heat tolerant lentils contain higher sucrose level [47] and heat-sensitive cultivars have lower sucrose and hexose contents than heat-tolerant cultivars under heat stress in chickpea plants [48], suggesting that a high CO_2_ level contributes to tolerance against high-temperature stress. Our results showed that global warming had no (Williams 82) or a negative (IT13414) effect on seed weight, in spite of the increased height, increased pod numbers, and increased seed numbers in our conditions (Figure S3), suggesting that there is a tradeoff between increased seed numbers and limited nutrient availability. However, the investigations of soybean yield during the last 60 years in the USA have revealed that the constant increment of CO_2_ concentration in the long term may have a negative impact on crop productivity, as a result of intensive crop cultivation and/or cultivar traits such as N_2_ fixation [49].

**Figure 4:**
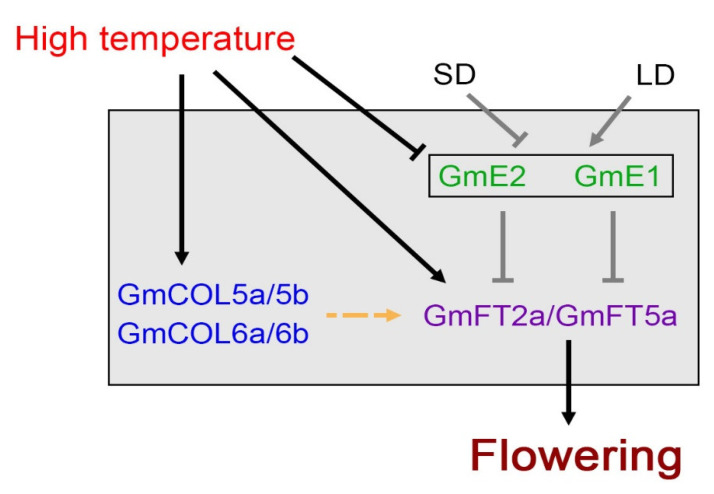
A proposed flowering model under global-warming conditions. High temperature promotes flowering through the transcriptional regulation of components in a photoperiodic pathway by repressing the expression of soybean floral repressors *E* loci genes (*GmE1* and *GmE2*) but activating the expression of floral activators *GmFTs* (*GmFT2a* and *GmFT5a*) as well as *GmCOLs* (*GmCOL5a/5b* and *GmCOL6a/6b*). Arrows and T-shaped symbols represent activation and inhibition of gene expression, respectively; the dotted line indicates a prediction that GmCOLs activates *GmFT2a* and *GmFT5a* expression. Grey lines are well approved from other studies and black lines are what we found in this study.

Flowering time is determined by a complex signaling network regulated by both endogenous genetic makeups and environmental stimuli, such as photoperiod and temperature [15,50,51,52]. Aside from cases of vernalization in winter crops, temperature affects flowering time by controlling the rate of development [53]. Long-term observation of over 400 flowering plants showed that global warming caused early-flowering times [54,55]. Here, we showed earlier flowering in both soybean cultivars, Williams 82 and IT13414 (Figure S3f), due to high-temperature conditions, as is shown in another legume plant, the chickpea [56]. In Figure 4, this is summarized in a working model.

The flowering network in *Arabidopsis* is one of the most well-studied flowering pathways, consisting of multiple subsets of endogenous and exogenous signaling [57]. In contrast to the model plant *Arabidopsis*, our knowledge of flowering time control of crop species by environmental factors such as high temperature is limited by our understanding on the molecular level. In soybean plants, genetic analyses showed that a number of *E* loci are important regulators for flowering and maturity, in particular under LD conditions [14,58]. Furthermore, the information of whole-genome sequences and molecular genetic studies has annotated many flowering genes responding on the photoperiodic pathway [14,16,20,21], such as *GmFTs* and *GmCOLs* (Figure 4, grey line on grey box).

In LD conditions, soybean plants delay flowering through the suppression of *GmFT2a* and *GmFT5a* expression by *E1* and *E2*; however, in floral inductive SD conditions, the expression of *E1* and *E2* is suppressed and the relieved *GmFT2a* and *GmFT5a* suppression induces flowering [20,22,23]. High-temperature conditions induced the expression of floral activators, *GmFT2a* and *GmFT5a* (Figure 1 and Figure 4), and suppressed their upstream negative regulators, *E1* and *E2,* expressions in a day length-independent manner (Figure 2 and Figure 4). Our results suggest that high temperature induces soybean flowering by affecting the activities of components in photoperiodic pathway, and moreover, the elevated temperature has a stronger effect on floral initiation than day length changes.

CO is well identified as a central controller in photoperiod-dependent flowering in *Arabidopsis* [29,52]; however, the roles of legume *CO-Like* genes (*COLs*) in flowering are suggested to be different from those of CO in *Arabidopsis* [30]. It is difficult to predict regulatory modules of *GmCOL5a/5b* and *GmCOL6a/6b*, or whether these *GmCOL* genes function in flowering promotion or not. Up-regulation of *GmCOL5a/5b* and *GmCOL6a/6b* under high-temperature conditions (Figure 3 and Figure 4) indicated that their expressions showed a positive relationship with the expressions of *GmFT2a* and *GmFT5a*, which further suggests that these *GmCOLs* are involved in high-temperature response. Recently, Zhang et al. (2020) reported that cool temperature (18 °C) delays flowering and up-regulates *GmCOL2b* expression at the fourth trifoliate leaf (V4) stage [59], suggesting that GmCOL2b works as a flowering repressor upon cool ambient temperatures. However, *GmCOL1a/1b* and *GmCOL2a/2b*, which are the best-known *GmCOL* genes in soybean plants, were, unexpectedly, not changed in response to high temperature (Figure S5). It is not clear that *GmCOL5a/5b* and *GmCOL6a/6b* directly target *GmFT2a* and *GmFT5a* like in *Arabidopsis*; however, we suggested that *GmCOL5a/5b* and *GmCOL6a/6b* would be candidates to regulate *FT* genes during high temperatures.

In conclusion, we created a working model for soybean growth and flowering under global warming conditions. High-temperature conditions promote flowering by changing floral gene expression in a day length-independent manner, i.e., through the down-regulation of repressor genes (*E1* and *E2*) and up-regulation of floral activator *FT* genes (*GmFT2a* and *GmFT5a*). In addition, although the molecular functions of *GmCOL5a/5b* and *GmCOL6a/6b* are unclear, we firstly showed that these *GmCOL* genes are involved in the regulation of soybean growth and development by responding to high-temperature conditions.

In this study, we primarily investigated floral pathway integrators, including FT and COL, to understand the acceleration of flowering in response to elevated temperatures using young soybean leaves. Because the leaf organ is a major sensor of temperature and photoperiod [60] and it is well known that the FT floral integrator is expressed in leaves, move to apical meristem, and contribute to reprogramming shoot apical meristem identity to floral meristem by activating meristem identity genes, such as *APETALA1* (*AP1*) and *LEAFY* (*LFY*) [15,20]. Testing the expression patterns of another floral integrator gene, such as *SUPPRESSOR OF OVEREXPRESSION OF CONSTANS* (*SOC1*), and floral meristem-identity genes, such as *AP1* and *LFY*, in the floral buds in response to high-temperature conditions would be an interesting avenue of study in the future.

## 4. Materials and Methods

### 4.1. Plant Materials and Growth Conditions

The two soybean cultivars, Williams 82 and IT153414, were used for gene expression analyses. Soybean seed-planted pots were individually placed in a growth chamber at both 20 °C and 30 °C under SD (12 h Light/12 h Dark) and LD (16 h Light/8 h Dark) conditions, and the trifoliate leaves were harvested at the V1 stage. To investigate soybean growth and development, we followed the method shown on the following soybean growth and development poster from K-state: (https://www.bookstore.ksre.ksu.edu/pubs/MF3339.pdf).

### 4.2. Semi-Quantitative Reverse Transcription Polymerase Chain Reaction (RT-PCR) and Quantitative Real Time PCR (qRT-PCR)

Total RNA was extracted from the first trifoliate leaves of three independent plants at the V1 stage using the LiCl method [61]. To remove genomic DNA contaminants, extracted RNA was treated with DNaseI (Sigma-Aldrich, St. Louis, MO, USA). Two microgram (µg) total RNA was used for cDNA synthesis using a RevertAid First Strand cDNA Synthesis Kit (Thermo Fisher Scientific, Waltham, MA, USA), according to the manufacturer’s protocol.

For the RT-PCR reaction, a 500 ng cDNA template was reacted with 5 units of Ex Taq polymerase (TaKaRa Korea Biomedical Inc., Seoul, Korea), 1X Ex taq buffer, 2.5 mM dNTP mixture, and 1 µmole of each gene-specific primers in 20 µL, according to the manufacturer’s protocol. The PCR was performed using the following conditions: 95 °C for 2 min for pre-denaturation, 30 or 35 cycles at 95 °C for 30 s, 55 or 58 °C for 15 s, and 72 °C for 30 s for 3 cycles. The RT-PCR products were analyzed by 1.5% agarose gel electrophoresis. The qRT-PCR analysis was performed using the QuantiSpeed SYBR No-Rox Mix (PhileKorea, Seoul, Korea), and the relative values of indicated gene expression were automatically calculated using the CFX96 real-time PCR detection system (Bio-Rad Laboratories, Hercules, CA, USA) by applying normalization of the expression of *GmPBB2*. The qRT-PCR was performed using the following conditions: 50 °C for 10 min, 95 °C for 10 min; followed by 50 cycles at 95 °C for 15 s, 58 °C for 15 s, and 72 °C for 15 s. The sequence and annealing temperature of gene specific primers used for RT-PCR and qRT-PCR analysis are listed in Supplementary Table S1.

### 4.3. Statistical Analyses

Statistical analyses in our experiments, including Student’s *t*-test, were performed using Microsoft Excel version 2016 program. The qRT-PCR analysis was performed in three independent experiments and the average values of 2^−ΔΔCT^ were used to determine the differences. Data and error bars are indicated as means ± standard deviation (SD).

## Data Availability

The data that support the findings of this study are available from the corresponding author upon reasonable request.

## Supplementary Materials

Supplementary materials can be found at https://www.mdpi.com/1422-0067/22/3/1314/s1.
