# Supplementary material for: High-Temperature Conditions Promote Soybean Flowering through the Transcriptional Reprograming of Flowering Genes in the Photoperiod Pathway
Source: Int J Mol Sci. 2021 Jan 28;22(3):1314. doi: 10.3390/ijms22031314 (PMC7865498; doi:10.3390/ijms22031314)
Supplement: Supplementary file 1 [file ijms-22-01314-s001.pdf]

Supplementary Materials

High-Temperature Conditions Promote Soybean Flowering through the Transcriptional Reprograming of Flowering Genes in the Photoperiod Pathway

Dong Hyeon No, Dongwon Baek, Su Hyeon Lee, Mi Sun Cheong, Hyun Jin Chun, Mi Suk Park, Hyun Min Cho, Byung Jun Jin, Lack Hyeon Lim, Yong Bok Lee, Sang In Shim, Jong-Il Chung, and Min Chul Kim

Supplementary Materials:

Figure S1. The open-top Climatron chamber

Figure S2. The vegetative growth comparison of William 82 and IT153414 at the open field and Climatron chamber.

Figure S3. Physiological characteristics of soybeans.

Figure S4. Expression analysis of soybean *E1* and *E2* homolog genes in Williams 82 and IT153414 cultivars under high-temperature conditions.

Figure S5. Expression analysis of soybean *CO-Like* (*GmCOL*) genes in Williams 82 and IT153414 cultivars under high-temperature conditions.

Table S1. Primer sequences used in this study

Experimental Method 1. Plant materials and environmental conditions for soybean growth conditions.

Experimental Method 2. Measurement of soybean physiological traits

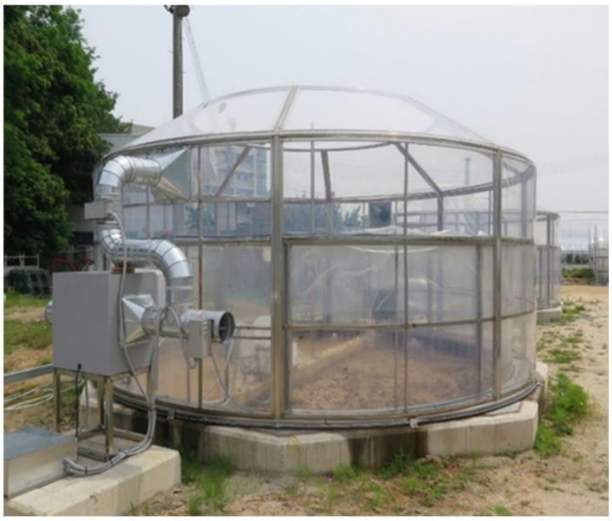

Figure S1. The open-top Climatron chamber. The chamber is cylindrical in shape with a bottom of 6 meter in diameter, an open-top of 3.4 meter in diameter and 30-degree, and a height of 3 meter. The wall is covered with Ethylene-Tetra Fluoro Ethylene copolymer (ETFE) with 93% transmission efficiency of light (Asahi Glass Co. Ltd, AGC inc. Tokyo, Japan). Temperature and CO_2 sensor is used a dual wavelength non-dispersive infrared technology (NDIR) which is manufactured by E+E Elektronik (Engerwitzdorf, Austria).

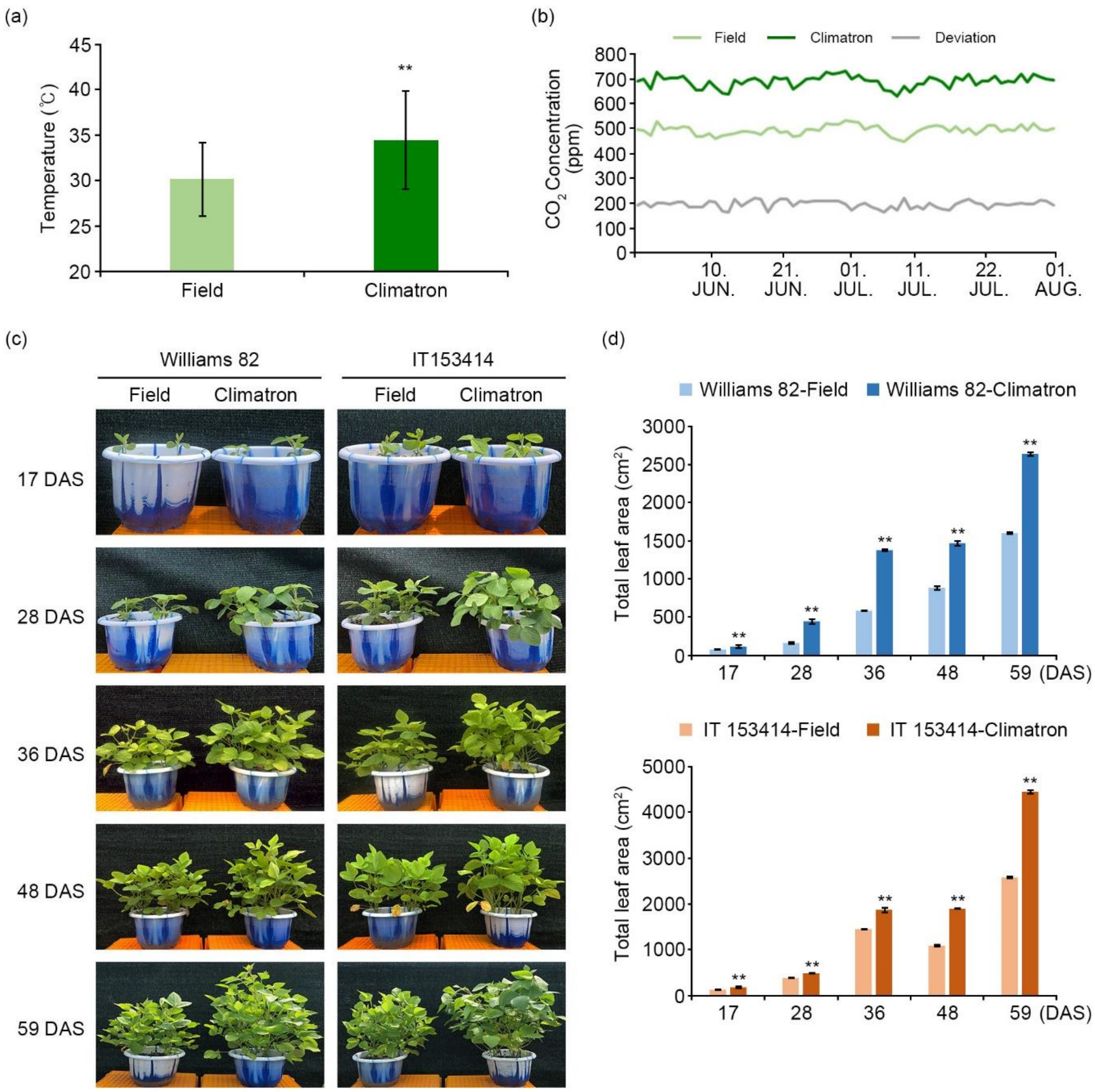

Figure S2. The vegetative growth comparison of William 82 and IT153414 at the open field and a Climatron chamber. Comparison of the effects of the temperature (℃) (a) and CO_2 concentration (ppm) (b) in an open field and an open-top Climatron chamber. Temperature and CO_2 concentration are shown as average values from daily measurements taken between May 1 to August 1, 2019. Bright green, field; Dark green, Climatron; Gray, the gap between field and Climatron. (c) The vegetative growth of William 82 and IT153414. Photographs were taken at indicated days after sowing (DAS) in a field (left) and in a Climatron chamber (right). (d) Total leaf area of Williams 82 and IT153414 grown in the open field and a Climatron chamber. Leaf area from (c) photos was measured using an Image J software (https://imagej.nih.gov/ij/download.html). The quantitative value indicated means ± SD of six individual plants. Blue, Williams 82; Orange, IT153414; Bright color, field; Dark color, Climatron. Asterisks represent significant differences between the open-top Climatron chamber temperature and field conditions (*, $p < 0.05$; Student's *t*-test). These observations were replicated in 2019 and 2020 with similar results.

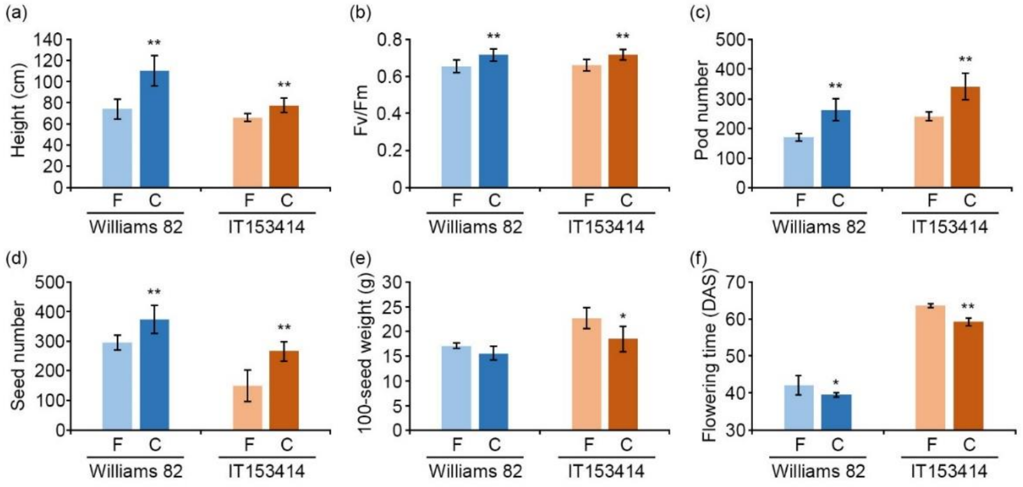

Figure S3. Physiological characteristics of soybeans. (a) Height, the maximum length. (b) Photosynthesis efficiency (F_v/F_m). Values were measured for the youngest trifoliate leaf using an OS30p+ chlorophyll fluorometer. (c) Pod number, (d) Seed number, and (e) 100-seed weight. (c–e) The number of pods (c) and seeds (d), and 100-seed weight (seed index, e) were measured during harvesting. (f) The flowering time in the field and Climatron. Flowering time was determined by counting the number of days from sowing to days at the first flower emerging in individual Williams 82 and IT153414 cultivars (DAS, n = 10). Bright color, field; Dark color, Climatron; Blue, Williams 82; Orange, IT153414. The quantitative values indicate means ± SD of 2019 summer and three independent experiments during 2019 and 2020 with similar results. Ten plants were used for the statistical analysis of these biometrics. Asterisks represent significant differences from the value in the field (*, $p < 0.05$; **, $p < 0.01$; Student's *t*-test).

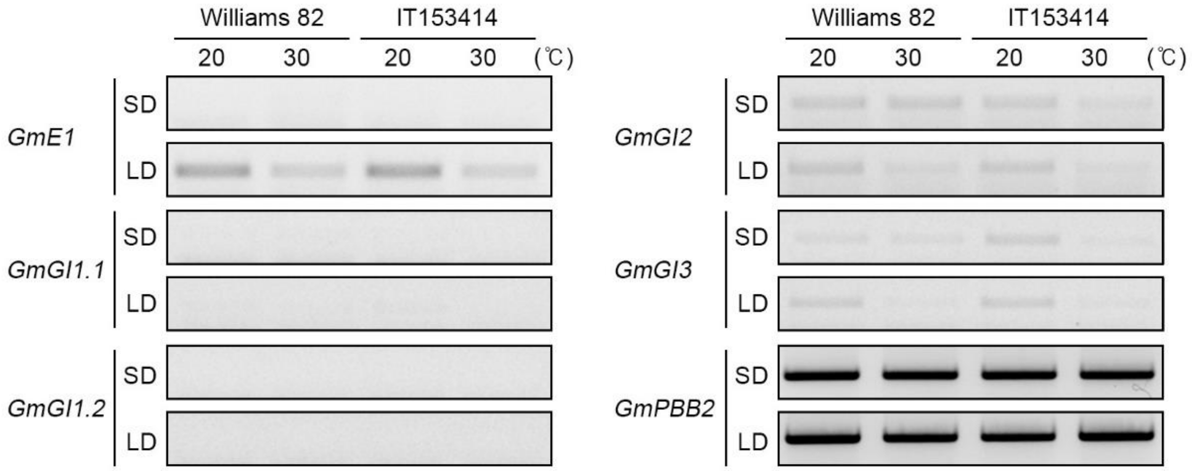

Figure S4. Expression analysis of soybean *E1* and *E2* homolog genes in Williams 82 and IT153414 cultivars under high-temperature conditions. Reverse transcription PCR (RT-PCR) was performed using gene-specific primers of *E1* and *E2* homolog. The amplicons were loaded at 1.5% agarose gel and analyzed transcript abundance. The expression of *GmPPB2* was used as a loading control. The RT-PCR analysis were performed in three independent replicates with similar results.

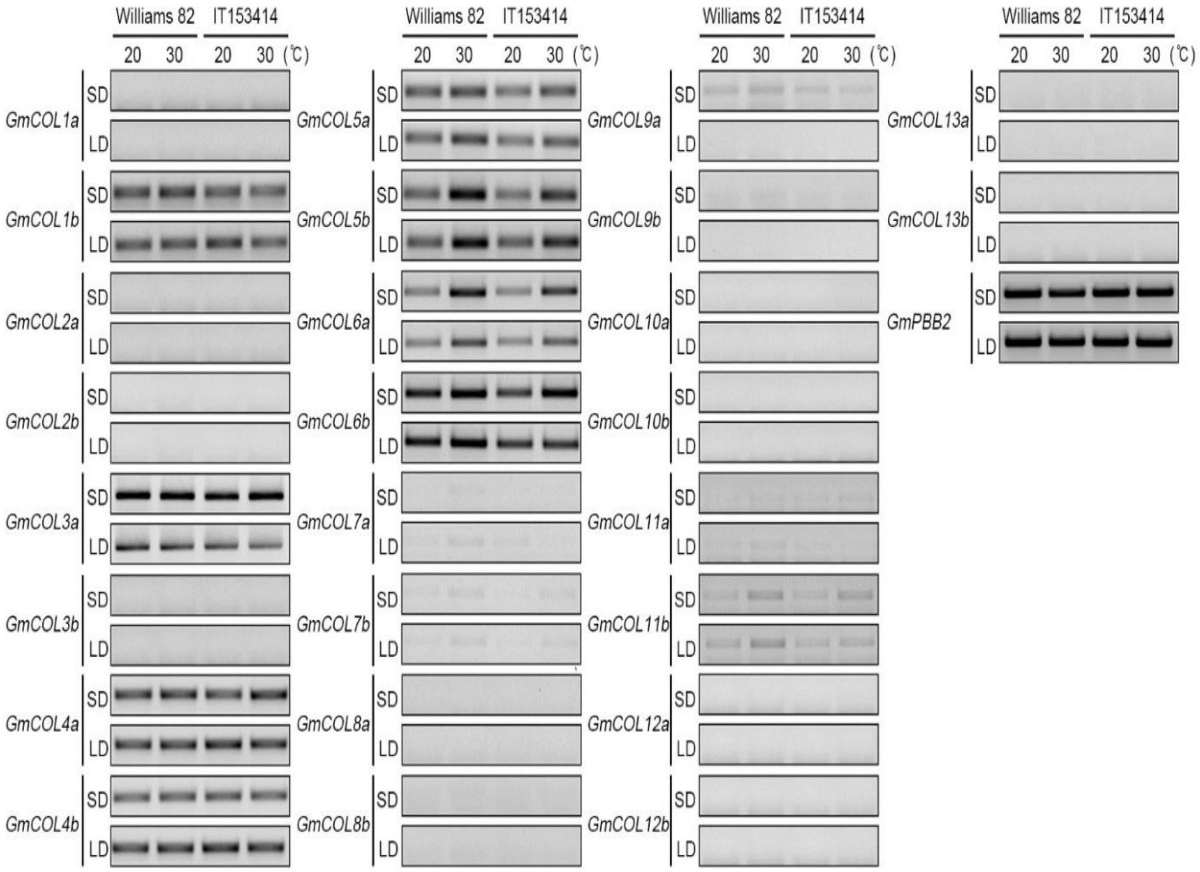

Figure S5. Expression analysis of soybean *CO-Like* (*GmCOL*) genes in Williams 82 and IT153414 cultivars under high-temperature conditions. Reverse transcription PCR (RT-PCR) was performed using *GmCOL* gene-specific primers. The amplicons of *GmCOL* genes were loaded at 1.5% agarose gel and analyzed transcript abundance. The expression of *GmPPB2* was used as a loading control. The RT-PCR analysis were performed in three independent replicates.

Table S1. Primer sequences used in this study

Gene	Primer	Direction	Sequence (5′→3′)	Tm (℃)
GmE1 (Glyma.06G207800)	MG-3286	Forward	5′-GGAAAGGTGATGAGATCGGA-3′	55.8
	MG-3287	Reverse	5′-GGTTGAAGTACACGCTATTGC-3′	56.7
GmGI1.1 (Glyma.20G170000.1)	MG-1083	Forward	5′-CCATTTGGGAAGCTGCTTATGG-3′	58.9
	MG-1085	Reverse	5′-GGAGGCAGACCCCACACAATAG-3′	61.7
GmGI1.2 (Glyma.20G170000.2)	MG-1642	Forward	5′-GATGGAGAAGCTTGCACTTTACCACAG-3′	63.2
	MG-1609	Reverse	5′-GAGCCTTAAACAGACCATATATCCACC-3′	58.4
GmGI2 (Glyma.10G221500)	MG-1290	Forward	5′-AACCCCACTACAGCCTCCCGTG-3′	65.2
	MG-1291	Reverse	5′-TGTACGAAGTTCTGCCACGGCC-3′	64.3
GmGI3 (Glyma.16G163200)	MG-1237	Forward	5′-ATTGAAGCCATCTTCTGTTGGCACG-3′	63.3
	MG-1238	Reverse	5′-GGTACAACAGATGTCTGCCCAGGGG-3′	65.8
GmFT2a (Glyma.16G150700)	MG-1117	Forward	5′- ATGGGGATTCATCGTTTGGTG-3′	57.6
	MG-1118	Reverse	5′- TGAAACCCTCAACGAAGCTTATACTAC-3′	60.1
GmFT5a (Glyma.16G044100)	MG-1127	Forward	5′-AGAGACACTGTCATCACCCCAGAATGG-3′	64.9
	MG-1128	Reverse	5′-GCTGGAGTAAGGCATCCAAGAATCTTC-3′	62.7
GmCOL1a (Glyma.08G255200)	MG-1039	Forward	5′-GATGTTGGATGGAGAAGCAACAATGGG-3′	63.5
	MG-1559	Reverse	5′-GTAGTAGCAACAGAAGCAAAGTGGTGG-3′	63.2
GmCOL1b (Glyma.18G278100)	MG-1073	Forward	5′-TATCAAGAGTGCTACTGTCCCTAACACC-3′	62.7
	MG-1074	Reverse	5′-GCTGAAGCGAAGTGATTATTATCACCACAAG-3′	63.6
GmCOL2a (Glyma.08G255200)	MG-1071	Forward	5′-CTCTTCGGGGAACCAGAGCATG-3′	62
	MG-1072	Reverse	5′-GCTGTAATTCTGCTGGTGCTGATC-3′	61.3
GmCOL2b (Glyma.18G278100)	MG-1069	Forward	5′-CTTCAGGGAACCAGAGCACAATCAC-3′	62.8
	MG-1558	Reverse	5′-CCACTACATAGTTCTGAGGGACAGTGTTGTAA-3′	64.5
GmCOL3a (Glyma.04G058900)	MG-1550	Forward	5′-GTTTGCGAAACGCACTGACC-3′	60
	MG-1551	Reverse	5′-CGAGGTGCTATTAGCATCGTTC-3′	58.3
GmCOL3b (Glyma.06G059600)	MG-1552	Forward	5′-GTTCGCGAAACGCACTGATG-3′	59.9
	MG-1553	Reverse	5′-CACGGCAGTCATAGCCGAACA-3′	62
GmCOL4a (Glyma.13G093800)	MG-1554	Forward	5′-GTCGGACATGTCGTATTCGAG-3′	57.7
	MG-1555	Reverse	5′-ATTCATGCACCCATCGTCTC-3′	57.1
GmCOL4b (Glyma.17G066600)	MG-1556	Forward	5′-CTTTTGGTCGGAACAGTTCGG-3′	58.8
	MG-1557	Reverse	5′-TTCTAAGATTCATGCACTCCATCGT-3′	59.4
GmCOL5a (Glyma.07G091400)	MG-2322	Forward	5′-ATATACAATGAAGTGAAGGACGAGGTTGTA-3′	61.2
	MG-2323	Reverse	5′-CTGATTCTTTCACGTGCTCATCATC-3′	59.5

GmCOL5b	MG-2324	Forward	5′-TACAATGAAGTGAAGGACGAGGTTAAT-3′	59.5
(Glyma.09G184600)	MG-2325	Reverse	5′-GAAGCACTAGTTCTGATCCTTTCTTCA-3′	60
GmCOL6a	MG-2326	Forward	5′-CAAAAGTAACCACCACGCACGCA-3′	63.7
(Glyma.05G233700)	MG-2327	Reverse	5′-TTATTCCAATGAAAGGCATCATCAAAAAC-3′	59.1
GmCOL6b	MG-2328	Forward	5′-ATCCTCTAAGGCAACCACAACCACT-3′	63
(Glyma.09G184600)	MG-2329	Reverse	5′-CTTATTTCATCGTCTTCTTTGTCAAGCC-3′	60.3
GmCOL7a	MG-1927	Forward	5′-TGATGAGAACGAGGAGCAGCTTC-3′	61.6
(Glyma.10G274300)	MG-1928	Reverse	5′-CACCAGAGGGCTCTCTTCCTGA-3′	62.1
GmCOL7b	MG-1925	Forward	5′-GATGAGAATGAGGAGCAGCTCG-3′	59.9
(Glyma.20G115600)	MG-1926	Reverse	5′-CCATCAGAGGGCTCTCTTCCTCT-3′	61.9
GmCOL8a	MG-2308	Forward	5′-GGTACCTCACCGTCTTCTTCGATG-3′	61.8
(Glyma.02G223700)	MG-2309	Reverse	5′-CACATTCTGCCCTTCTGGAGGAATG-3′	62.6
GmCOL8b	MG-1562	Forward	5′-GGTACCTCACCATCTTCTTCGACA-3′	60.9
(Glyma.14G190400)	MG-1563	Reverse	5′-TCTGGAGAAGCCCAAGCATTC-3′	59.4
GmCOL9a	MG-2310	Forward	5′-GTTATCACAAAGTGCCCCCTTCAGG-3′	62.8
(Glyma.13G009300)	MG-2311	Reverse	5′-TGTCAAAGCCAAATATTACAATTTACAACC-3′	59.1
GmCOL9b	MG-2312	Forward	5′-TCCAAGAATTCAAAAAGCCTTCTTCT-3′	58.7
(Glyma.20G060400)	MG-2313	Reverse	5′-TTGTGTGCTATACATGTCAAAGGTAAGAG-3′	60.6
GmCOL10a	MG-2314	Forward	5′-CTGCATTGGGACATAGGCGTG-3′	60.7
(Glyma.12G196100)	MG-2315	Reverse	5′-ATTTGATGGAATGATTTGAGACTGCC-3′	59.4
GmCOL10b	MG-1560	Forward	5′-CTGCATTGGGACATAGGCGCC-3′	63.1
(Glyma.13G306400)	MG-1561	Reverse	5′-TGAGACTGATCCATCCGAGGTTGA-3′	61.9
GmCOL11a	MG-1601	Forward	5′-GCCATAGCTATGAGTGGTGAAG-3′	57.6
(Glyma.03G209800)	MG-1602	Reverse	5′-AAGCCCTTCAATTTCTCCAC-3′	54.9
GmCOL11b	MG-1599	Forward	5′-ATTCATAGCTATGAGTGGTGCTG-3′	57.4
(Glyma.19G207100)	MG-1600	Reverse	5′-AGCCCTTCAATCTCGCTGG-3′	58.8
GmCOL12a	MG-2316	Forward	5′-CAGAGAACATGTGACTATTGCGGGAG-3′	62.5
(Glyma.02G152900)	MG-2317	Reverse	5′-CCTCATTTGAAAGCAGAGATTTTTCACTG-3′	60.8
GmCOL12b	MG-2318	Forward	5′-GAGAACATGCGACTATTGTGGGGA-3′	61.7
(Glyma.10G021400)	MG-2319	Reverse	5′-CCTCATTTGAAAGCAGAGATTTTTCAGAA-3′	60.1
GmCOL13a	MG-1603	Forward	5′-GACCAGATTCCGCTAAACTATGCTTGC-3′	63.4
(Glyma.16G050900)	MG-1604	Reverse	5′-GTACACCTCATCGCGACCGCC-3′	64.7
GmCOL13b	MG-2320	Forward	5′-GAGCTGATTCCGCTAAACTCTGTTTAG-3′	61.1
(Glyma.19G099700)	MG-2321	Reverse	5′-ATAAGCAAGGAGGTGAACGGCG-3′	62.2

GmPBB2	MG-1097	Forward	5′-TGCCGAAGAAACGCAATGCTTCAA-3′	63.6
(Glyma.14G014800)	MG-1098	Reverse	5′-TGCAGCAAGTGAACCTGATCCCAT-3′	63.7

Experimental Method 1. Plant materials and environmental conditions for soybean growth.
The two soybean cultivars, Williams 82 and IT153414, were used for plant physiological analyses. Soybeans were grown after sowing two seeds in a pot filled with a 20:1 ratio of Bio Bed Soil No. 2 (FarmHannong Co., Seoul, Korea) and Humic Rice Nursery Bed Soil No. 1 (Punong. Co., Gyeongju, Korea) in the field and Climatron. Growth of underground parts was controlled due to cylindrical pots, which were 23 cm in height and had an inner diameter of 30 cm. For the analysis of physiological phenotypes, pots were placed in the open-top Climatron chamber and in the open field (Figure S1), and each pot was used as an individual sample (n = 10).

Experimental Method 2. Measurement of soybean physiological traits.
To determine flowering time, the number of days after sowing was recorded as the appearance of the first flower in a field and Climatron chamber. For the height, maximum stem length was measured using a straight ruler when soybean growth stopped and leaves started to fall. The chlorophyll fluorescence (F_v/F_m) was measured using the OS30p+ chlorophyll fluorometer (Opti-Sciences, Inc., NH, USA) after dark adaptation, in which 7th trifoliate leaves were clipped for 30 min on the middle leaflet in the V7 stage (a vegetative stage). Next, the number of pods and seeds were counted from both the soybeans plants grown in the field and Climatron chamber. For the 100-seed weight, the weights of 100 randomly chosen seeds were measured.
